# Integrated computational approaches to screen gene expression data to determine key genes and therapeutic targets for type-2 diabetes mellitus

**DOI:** 10.1016/j.sjbs.2022.02.004

**Published:** 2022-02-10

**Authors:** Fahad A. Alhumaydhi

**Affiliations:** Department of Medical Laboratories, College of Applied Medical Sciences, Qassim University, Buraydah 52571, Saudi Arabia

**Keywords:** Type-2 diabetes mellitus, Differently expressed genes, Transcription factors, Gene expression omnibus, Molecular dynamics simulation

## Abstract

There is a rapid rise in cases of Type-2-diabetes mellitus (T2DM) globally, irrespective of the geography, ethnicity or any other variable factors. The molecular mechanisms that could cause the condition of T2DM need to be more thoroughly analysed to understand the clinical manifestations and to derive better therapeutic regimes. Tools in bioinformatics are used to trace out key gene elements and to identify the key causative gene elements and their possible therapeutic agents. Microarray datasets were retrieved from the Gene expression omnibus database and studied using R to derive different expressed gene (DEG) elements. With the comparison of the expressed genes with disease specific genes in DisGeNET, the final annotated genes were taken for analysis. Gene Ontology studies, Protein–protein interaction (PPI), Co-expression analysis, Gene-drug interactions were performed to scale down the hub genes and to identify the novelty across the genes analysed so far. *In vivo* and *invitro* analysis of key genes and the trace of interaction pathway is crucial to better understand the unique outcomes from the novel genes, forming the basis to understand the pathway that ends up causing T2DM. Afterwards, docking was executed enabling recognition of interacting residues involved in inhibition. The complex CCL5-265 and CD8A-40585 thus docked showed best results as is evident from its PCA analysis and MMGBSA calculation. There is now scope for deriving candidate drugs that could possibly detect personalized therapies for T2DM.

## 1 Introduction

Diabetes mellitus is a disorder causing blood glucose levels to be abnormally high. Blood glucose is the primary source of energy and accumulates in the blood from dietary intake. Insulin, a hormone made by the pancreas, helps blood glucose to enter cells to be used up as energy (Arif et al., 2021). Diabetes is an increasingly occurring disorder these days and its prevalence is growing worldwide and has reached epidemic proportions in many developing and most developed countries (Dedov et al., 2016). Further, it is also found out to be the most complex multifactorial disorder that could be an end point caused in combination by both genetic and environmental elements or individually (Dong et al., 2019, Sudesna et al., 2017). The disorder is primarily characterized by the deficiency of insulin production in the body and thereby resulting in uncontrolled hyperglycemia. It is roughly estimated that every second person suffers from diabetes of variable stages and complexities out of which Type-2-diabetes mellitus (T2DM) is one among the three most chronic metabolic disorders, threatening all kinds of population globally (Carey et al., 2004). T2DM has for itself about 90–95% share of the total cases in diabetes. These individuals do not need insulin treatment to survive often throughout their lifetime. Autoimmune destruction of β-cells do not occur due to T2DM with its specific etiologies still unknown (Association 2009). After the year 2015, it is estimated that about 90.5% occurrences of diabetic conditions in patients tested or otherwise, is a derivative outcome of T2DM and is notoriously common among ethnicities in undeveloped geographies across the globe. The average incidence and prevalence of T2DM is visibly more in females versus males regardless of their age group (Dedov et al., 2016). It is also very commonly seen in women with prior Gestational diabetes mellitus (GDM) and in individuals with hypertension or dyslipidemia. Only that, its intensity and frequency may vary with populations and subgroups. It is also often associated with a strong, complex and unclear genetic predisposition (Association 2009).

The discovery of biomarkers which can potentially subside the pathophysiology of the disorder can be a good motive that could inspiringly lead to the discovery of more effective therapeutic options in future (Hsiang et al., 2015). Potential benefits of molecular biomarkers propound prospects to enhance the efficacy of diagnostic and therapeutic regimes to fight T2DM. Bioinformatics tools are popular today for all kinds of biological research streaks. Lately, there is an increasing emergence of sequencing technologies that equip researchers for their novel discoveries in the domains of both computational biology and molecular therapeutics (Chang et al., 2019). In the recent past, microarray technologies have spurred avenues for identification of pathological conditions that could have been potentially caused by the genes that are involved in the pathogenesis in the clinical manifestations with T2DM (Brechot et al., 2010).

The Medicinal plants possess secondary metabolites that are valuable sources for the treatment of many disorders and diseases (Ahmed et al., 2014, Tahir ul Qamar et al., 2019, Arif et al., 2021, ul Qamar et al., 2021). These herbs are cost effective, easily accessible and bear structural diversity to secondary metabolites (Amos et al., 2003, Khan et al., 2017). Therefore, several traditional plants have been investigated for their used as medicinal plants and gaining huge importance for its discovery as lead molecule (Ameer, 2011, Muneer et al., 2021).

There is not much substantiation that is currently available to validate the existence of disease-causing genes, and their elemental contribution to the incidence, prevalence or pathogenicity of T2DM. Integrative bioinformatic approaches were explored and understood to trace out the pathogenetically functional genes as derivative negotiators that could turn down the progression of T2DM. Differential Gene Analysis (DEG) is performed with Gene ontology analysis (Gene ontology and reactome). Pathways in a reactome are placed in a hierarchy, so as to group up details of pathways for translation, protein folding and post-translational modification into larger domains of biological function like protein metabolism. This arrangement enables Gene Ontology (GO) which is nothing but a biological process hierarchy (Fabregat et al., 2016).

Protein-Protein Interactions (PPI) is used to list out the hub genes that could be causing the condition and co-expression network was used to validate the listed genes by a heat map considering the co-regulation scores of these genes (Chang et al., 2019). Nonetheless, investigative efforts on drug-gene interactions in our work, potentially inspire the research activities to identify therapeutic drug candidates for T2DM (Donadon et al., 2008). The STRING database collects, scores and integrates all openly available information on protein–protein interaction, so as to complement with computational predictions. This exercise helps us build an objective and the most comprehensive global network, inclusive of both physical and functional interactions (Szklarczyk et al., 2021).

In this study, T2DM targeting genes were analyzed for Gene Ontology studies, Protein–protein interaction (PPI), Co-expression analysis and Gene-drug interactions. It was subsequently followed by exploring interaction pathway study. The screened the top-ranked complex CCL5-265 and CD8A-40585 after docking (Altharawi et al., 2021) was further chosen for MD simulation (Tahir ul Qamar et al., 2021) which demonstrated outcomes within the reliable range of parameters that signifies the interaction outcomes scrutinizing the study. This paper describes study of Integrated computational approaches to screen gene expression data to determine key genes and therapeutic targets for type-2 diabetes mellitus using techniques such as gene expression analysis, ontology, Protein-protein Interaction (PPI), modeling and docking.

## 2 Materials and methods

**Fig. 1:**
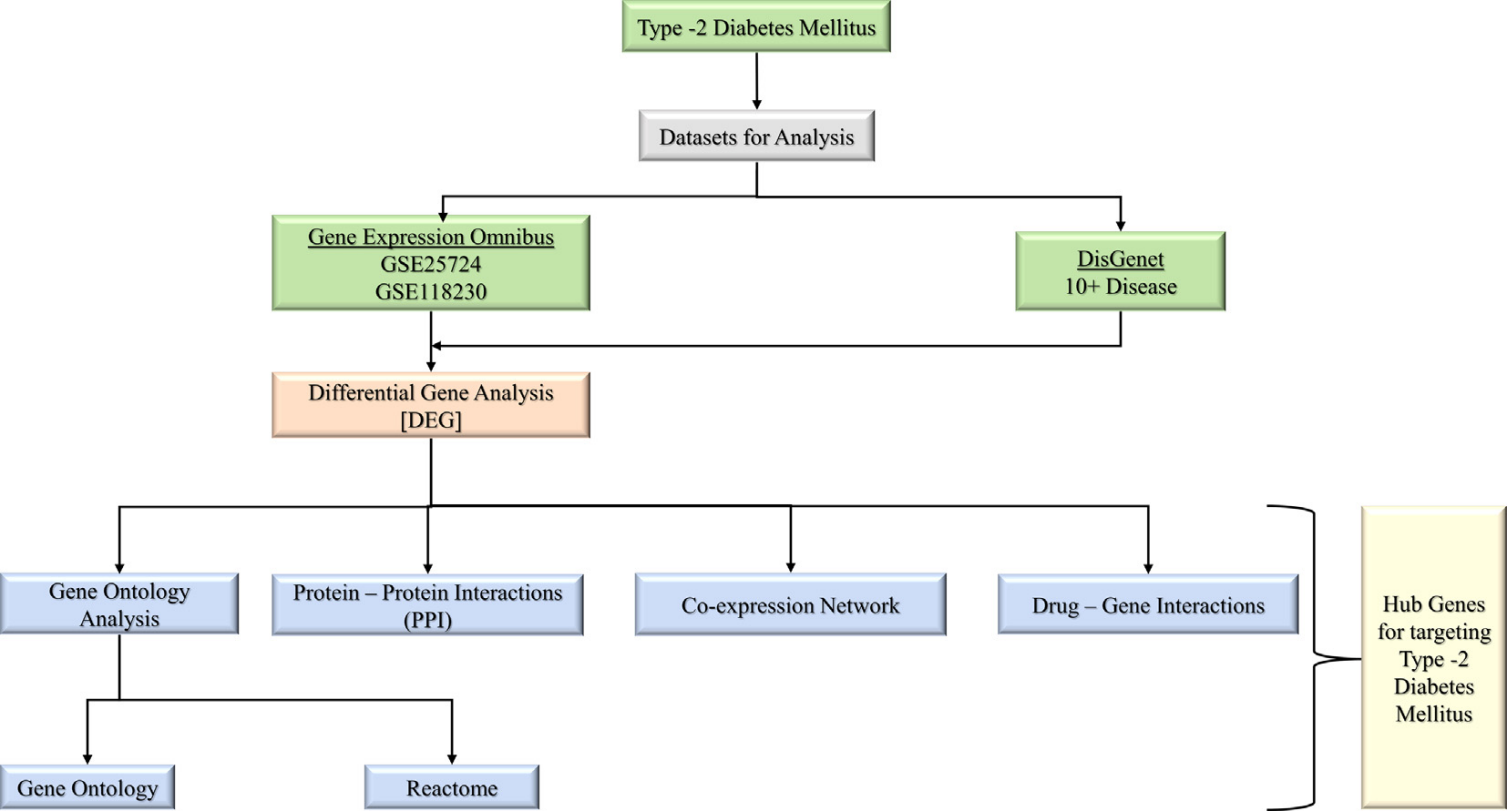
Schematic representation of the tools and methods used in the study.

The approaches and steps used in the study are briefly outlined in Fig. 1.

### 2.1 Data sourcing

An open source database, NCBI-GEO (https://www.ncbi.nlm.nih.gov/geoprofiles/) contains gene expression datasets (Barrett et al., 2009). Two gene expression profile datasets of T2DM (GSE25724 and GSE101931) were sourced from peripheral blood mononuclear cells (Berry et al., 2018). More information about sample datasets is given in Table 1 below.

**Table 1:** Sample datasets from GEO database.

GEO IDs	Source	Experiment type	Year of research
GSE25724	Human islets from Pancreas	Expression profiling by array	2017
GSE101931	Human islets from Pancreas	Expression profiling by array	2018

The retrieved microarray datasets were analyzed using GEO2R to identify significant up-regulation and down-regulation of genes. On the other hand, DisGeNET was used to find disease specific genes through disease keyword mapping as follows: “Name: Type-2-diabetes mellitus in obese; CUI: C0271638:: Name: Diabetes Mellitus, Non-Insulin-Dependent; CUI: C0011860:: Name: Type 2 diabetes mellitus in nonobese; CUI: C1282951:: Name: Type-2-diabetes mellitus with periodontal disease; CUI: C2874119:: Name: Insulin treated type-2-diabetes mellitus; CUI: C0342266:: Name: Type-2-diabetes mellitus without complication; CUI: C0494290:: Synonym: Type-2-diabetes mellitus with unspecified complications; CUI: C1299614:: Synonym: Pre-existing type-2-diabetes mellitus; CUI: C0348921:: Name: Non-insulin dependent diabetes mellitus with unspecified complications; CUI: C1299614:: Name: Pre-existing diabetes mellitus, non-insulin-dependent; CUI: C0348921:: Synonym: Insulin treated non-insulin dependent diabetes mellitus; CUI: C0342266:: Synonym: Non-insulin dependent diabetes mellitus without complications; CUI: C0494290:: Name: Non-insulin dependent diabetes mellitus with unspecified complications; CUI: C1299614:: Name: Pre-existing diabetes mellitus, non-insulin dependent; CUI: C0348921:: Synonym: Insulin-treated non-insulin-dependent diabetes mellitus; CUI: C0342266:: Synonym: Non-insulin dependent diabetes mellitus without complications; CUI: C0494290”. The list of genes associated with these diseases were studied in comparison with the differentially expressed genes and the following workflow was designed to derive an overview on the top-down bioinformatics approach used to scale down the hub genes of T2DM.

### 2.2 Differential expressed genes (DEGs) markers

Differential gene expression is crucial for biological depiction of conditional differences between a healthy and a diseased status of the body (Sufyan et al., 2021). Differentially Expressed Genes (DEGs) for T2DM were pointed out using the GEO Database (https://www.ncbi.nlm.nih.gov/geo/), which is an interactive tool to analyze and compare the data of two or more sample groups under the similar experimental conditions (Barrett et al., 2012). Genes that fit the elemental criteria with adjusted P_value_ to be lesser than 0.05 (P_value_ < 0.05) were identified as DEGs. Genes that portray the features of up-regulation or down-regulation in T2DM were strategically identified by an online Venn diagram tool called Venny 2.1 (http://bioinfogp.cnb.csic.es/tools/venny/).

### 2.3 Functional analysis of DEGs

Functional enrichment analysis is used to determine classes of genes or proteins that are over-represented in a large group of genes or proteins and may have relations with disease phenotypes. Enrichment analysis of Gene Ontology (GO) was done (Griss et al., 2020) to predict the impending functions of the hub genes using an open-source web-based tool called Reactome (https://reactome.org/). Tri-level functional prediction analysis was performed on the DEGs using the Reactome tool to understand biological process (BP) functions. Generation of heat maps for the BP pathway was calculated by - log_10_ (P_value_) and a clustered bar chart of top 15 pathways were constructed.

### 2.4 Construction of PPI network and identification of hub genes

A protein–protein interaction (PPI) network systematically identifies disease-related genes from the connection between those genes with similar functions. The interactive networks of the well-known DEGs among all datasets were constructed using a web-based interactive search tool called STRING (https://string-db.org/) for those with interaction scores greater than 0.5. The network analysis was performed by the visualization of the PPI network and further understood by the tool Cytoscape 3.8.2 (Demchak et al., 2014).

### 2.5 Co-expression analysis

The 61 annotated genes were submitted to the STRING database for the analysis of Co-expression analysis. A complete table of co-expressed genes was retrieved from STRING and a grid network was created using Cytoscape 3.6.1. The genes with co-expression score > 0.5 were shortlisted and identified as hub genes. A heat map considering the co-regulation scores of these genes through ProteomeHD was created. The genes on the heatmap were identified as hub genes through co-expression validation.

### 2.6 Drug-gene interaction

A discovery of a drug-gene interaction does not necessarily indicate efficacy of any decided treatment regime with any drugs (Freshour et al., 2021). Drugs were only selected, purely on the basis of hub genes that are definitive drug targets with the help of their Drug gene interaction database (DGIdb) (https://www.dgidb.org/) [29]. The drugs that are used are also drugs that are approved by the Food and Drug Administration (FDA) with DrugBank sourcing.

### 2.7 Molecular docking and simulation of unique targets (CCL5 and CD8A)

Molecular docking and simulation studies were employed on the two selected unique targets such as CCL5 and CD8A. For this purpose, structure of CCL5 with PDB ID:6AEZ was retrieved from protein data bank database. Structure of CD8A was predicted through phyre2 protein fold recognition tool. A comprehensive library of 12,000 phytochemicals was docked with these two protein structures and top five compounds were banked on the basis of root mean square deviation (RMSD), energy function score (S_Score) and hydrogen bonding interaction (H-bond accepter or H-bond donor). MOE software was used to perform molecular docking. Before molecular docking, both the protein structures were minimized in a Molecular operating environment (MOE) by selecting the MMFF94x force field.

Furthermore, to validate the molecular docking results, molecular dynamics simulation of the one best compound for each protein was performed through Desmond (version 3.6 package). The solvent model “TIP3P” with orthorhombic boundary box was used. To neutralize the protein ligand complex system, OPLS-2005 force field was used by adding the Na^+^ Cl^-^ salt ions. Protein ligand complex was further minimized using the steepest method of maestro with LBFGS algorithms. For both protein ligand complexes MD simulation was carried at 100 ns to check the stability of the complexes.

## 3 Results

### 3.1 Differential expression analysis identifies overlaps between the genes

**Fig. 2:**
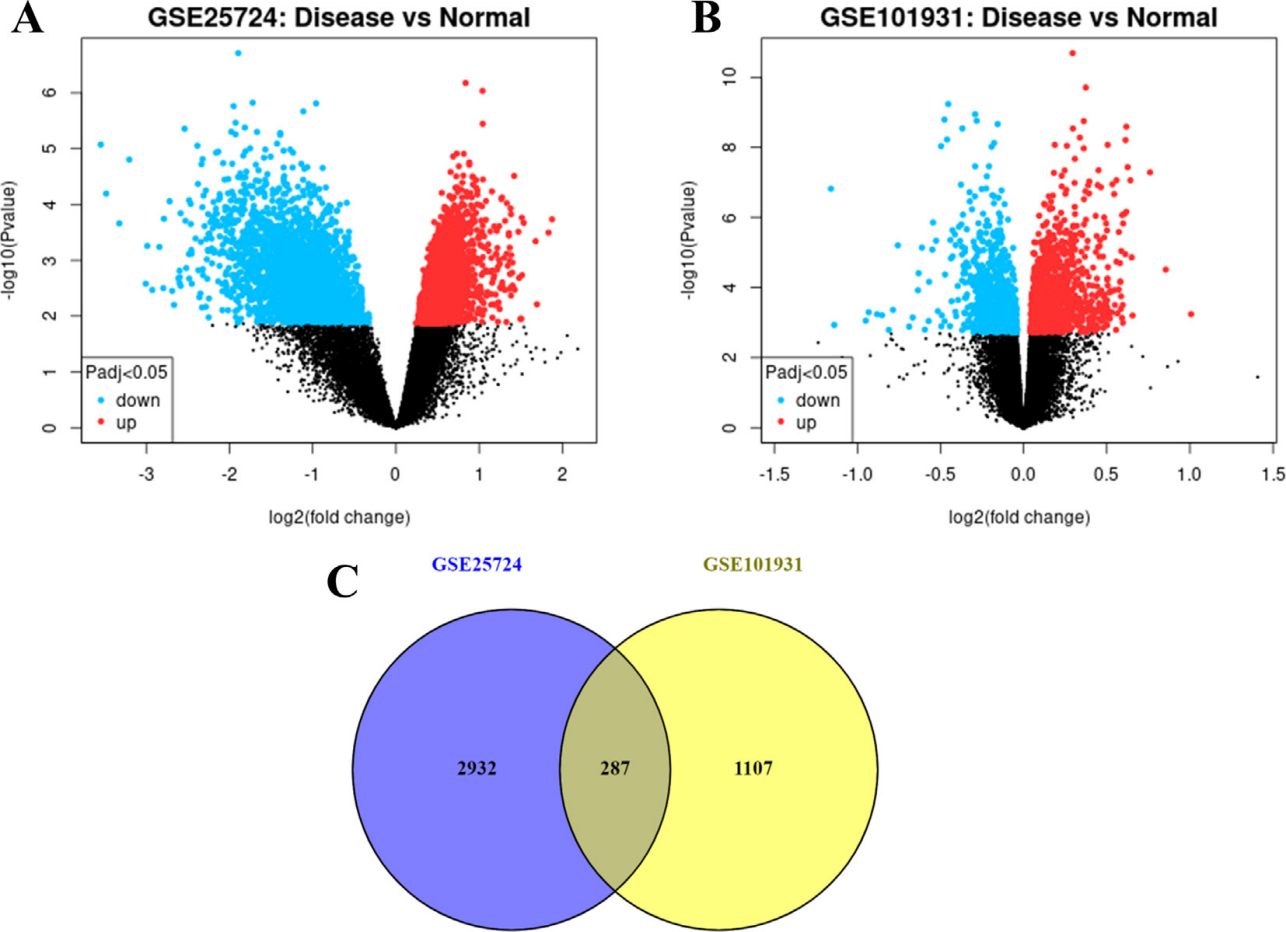
**Comparison of Curated Microarray Datasets. (A)** Volcano Plot of GSE25724. On comparison of Disease vs Normal genes, post-normalization, a total of 5949 genes were significantly expressed with 2453 and 3496 genes were downregulated and upregulated respectively. **(B)** Volcano Plot of GSE101931. On comparison of Disease vs Normal genes, post-normalization, a total of 1816 genes were significantly expressed with 798 and 1018 genes were downregulated and upregulated respectively. **(C)** Venn diagram of overlapping genes. The normalization of the genes yielded 5949 and 1816 expressed genes. However, considering the annotation of the gene, the final list of genes taken for overlaps were 3220 and 1395 on GSE25724 and GSE101931 datasets respectively. Out of these, 287 genes intersect in both datasets.

The microarray datasets taken from NCBI - GEO were analyzed using GEO2R. The parameters were set to default and the analysis was performed with p-value < 0.05 and the Benjamini & Hochberg for decreasing false discovery rate (FDR). Quantile normalization was performed, creating a volcano plot for the condition of disease vs normal. This analysis yielded 3220 and 1395 on GSE25724 and GSE101931 datasets after annotation respectively. Out of these, 287 genes intersect in both datasets (Fig. 2). This data is used for further disease annotation.

### 3.2 Curated data narrows the identification of hub genes

**Fig. 3:**
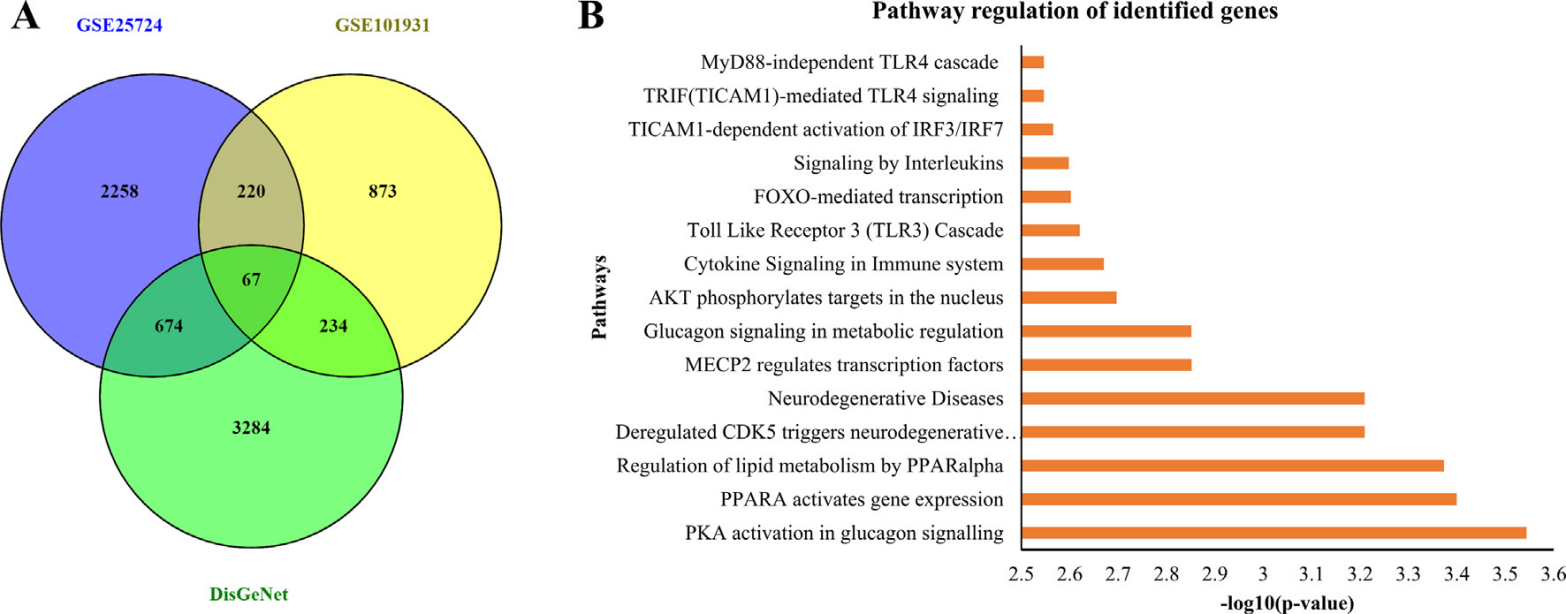
(**A**) The association between the microarray datasets and the DisGeNET dataset. (**B**) Gene ontology-based pathway mapping of the curated list of genes.

The datasets GSE25724 and GSE101931 had 3220 and 1395 differentially expressed genes respectively. This data was compared against the set of disease conditions in the DisGeNET database. Out of the 8296 genes found from the DisGeNET database, 67 genes were located to be expressed in the differentially expressed dataset. These 67 genes were curated and a final list of 61 genes for the comparison and identification of hub genes were used. The other 6 genes were synonymous to the list and were considered a duplicate. The associations of the above 3 datasets are explained using a Venn diagram below (Fig. 3A).

### 3.3 Gene ontology maps the associated pathways

With the list of genes, it is crucial to understand the pathways associated with them. This would pave the way in understanding the disease mechanism and the correlation of the identified genes to the diseases (Mi et al., 2019). Reactome and Gene Ontology databases were used to find the associated pathways of the 61 genes identified. Reactome produces more efficient pathway mapping than Gene Ontology. The former produced top pathways including activation pathways PKA, PPARA, transcriptional activation and expression, signaling pathways like glucagon signaling, cytokine and interleukin signaling. On the other hand, the top pathways of the latter included various response and regulation pathways rather than pathways specific to the disease mechanism. Majorly, the top 15 pathways were chosen based on higher -log_10_(p-value). The top 15 pathways had -log_10_(p-value) ranging from 2.56 to 3.57. The list of the pathways and its associated -log_10_(p-value) are provided in the clustered bar chart below (Fig. 3B). Also, when this was compared to the disease of interest type-2-diabetes mellitus, the genes were vast and variegated to link multiple pathway associations to understand disease mechanisms. The detailed table of the mapped pathways are provided in supplementary data.

### 3.4 Protein-protein interaction identifies the hub genes

**Fig. 4:**
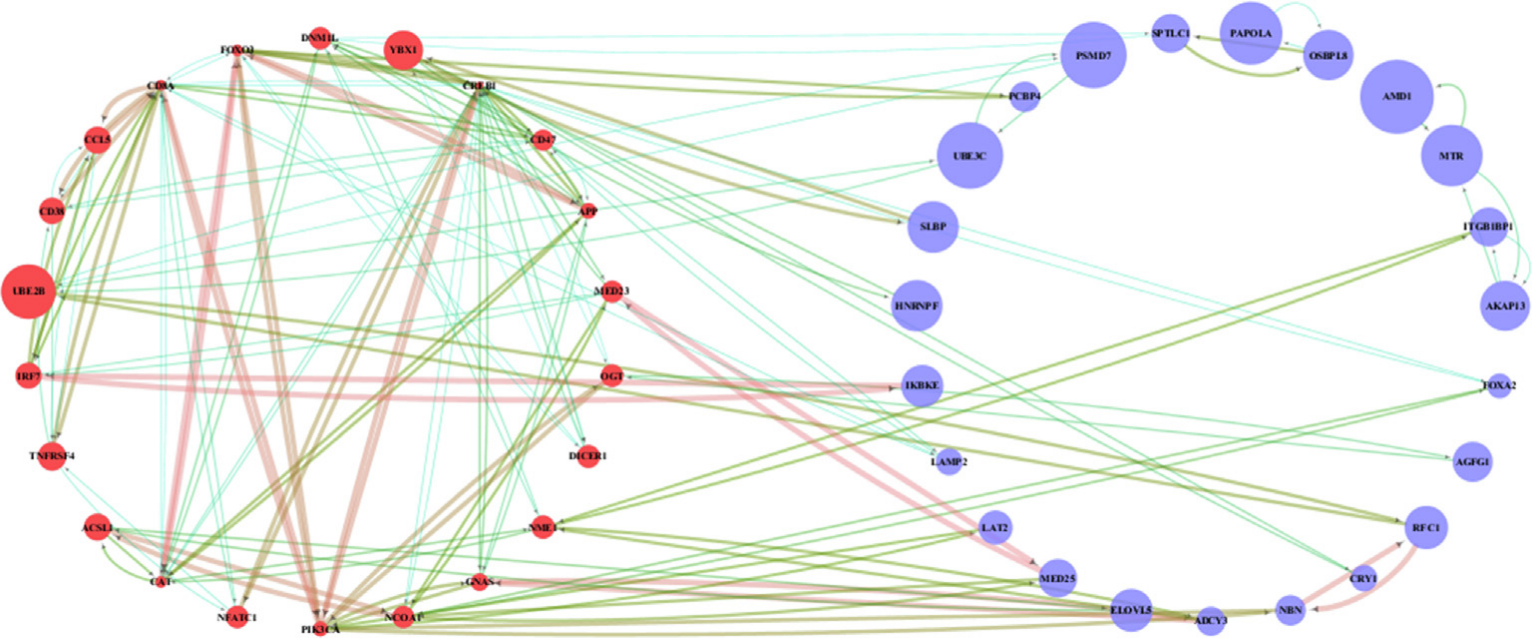
Identification of hub genes using STRING based protein interactions. The round is the gene where the red ones are the hub genes, and the blue ones are the interacting genes. The size of the genes is based on the average shortest path length and range between 2.36 and 6.82. Similarly, the edges were highlighted based on the interaction score and it ranges from 0.4 at the blue lines, 0.6 at the green to 0.9 at the red line. The complete table of interaction and network analysis is detailed in supplementary data.

The annotated 61 genes were mapped as protein-coding genes and were submitted to the STRING database for the identification of the interactions. A full STRING network with a moderate confidence score of 0.40 and the FDR as 0.05 was set and the final PPI network was retrieved. The table with the confidence score was uploaded to Cytoscape 3.6.1 and the network was analyzed. With the removal of isolated nodes, the final network yielded 45 nodes and 148 edges. The network was analyzed using the network analyzer unveiling the network parameters. The average number of neighbors was 3.2 and with the degree filter the nodes within the range of 3 to 13 out degree were separated and grouped based on the selected nodes. This yielded 22 nodes which were a part of the 61 submitted to the STRING. Also, the size of the nodes was subjected to the increasing average shortest path length and the size of the edges were subjected to the interaction score provided by the STRING database. The 22 source genes are shortlisted as hub genes (Genes colored in red in Fig. 4). The annotated forms of the identified hub genes are listed in the Table 2.

**Table 2:** Annotation of hub genes as protein coding genes in Uniport. In addition to this, the complete functional annotation of the genes is provided in the supplementary data.

Gene names	Uniprot Entry	Protein names
CREB1	P16220	Cyclic AMP-responsive element-binding protein 1
CD8A	P01732	T-cell surface glycoprotein CD8 alpha chain
PIK3CA	P42336	Phosphatidylinositol 4,5-bisphosphate 3-kinase catalytic subunit alpha isoform
CAT	P04040	Catalase
FOXO3	O43524	Forkhead box protein O3
APP	P05067	Amyloid-beta precursor protein
DNM1L	O00429	Dynamin-1-like protein
NCOA1	Q15788	Nuclear receptor coactivator 1
CD47	Q08722	Leukocyte surface antigen CD47
NME1	P15531	Nucleoside diphosphate kinase A
GNAS	P63092	Guanine nucleotide-binding protein G(s) subunit alpha isoforms short
IRF7	Q92985	Interferon regulatory factor 7
MED23	Q9ULK4	Mediator of RNA polymerase II transcription subunit 23
CD38	P28907	ADP-ribosyl cyclase/cyclic ADP-ribose hydrolase 1
CCL5	P13501	C-C motif chemokine 5
TNFRSF4	P43489	Tumor necrosis factor receptor superfamily member 4
DICER1	Q9UPY3	Endoribonuclease Dicer
UBE2B	P63146	Ubiquitin-conjugating enzyme E2 B
YBX1	P67809	Y-box-binding protein 1
ACSL1	P33121	Long-chain-fatty-acid--CoA ligase 1
OGT	O15294	UDP-N-acetylglucosamine--peptide N-acetylglucosaminyltransferase 110 kDa subunit
NFATC1	O95644	Nuclear factor of activated T-cells, cytoplasmic 1

This list of 22 genes includes variegated types including proteasome mediated genes, immunoregulatory genes, glycosylation-based genes and biosynthesis genes. This narrows down the list of the genes from the differentially expressed genes. However, this needs further validation and scaling down of the number of genes specific to Type-2 diabetes mellitus.

### 3.5 Co-expression analysis scales down the list of hub genes

**Fig. 5:**
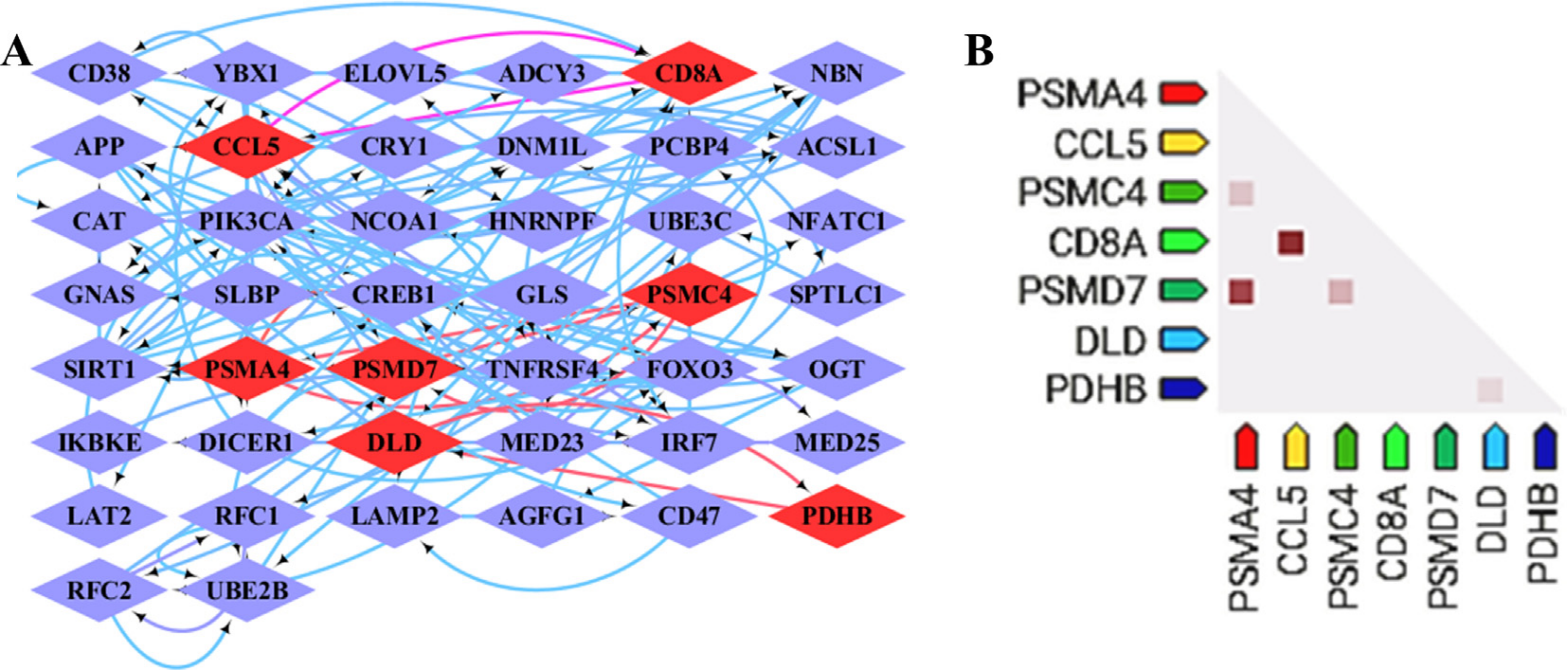
**Co-expression analysis of the annotated genes through the STRING database.** (**A**) Grid layout of the co-expressed gene network drawn in Cytoscape based on decreasing score. The edges were colored based on the co-expression score ranging from 0.05 in blue, 0.41 in pink to 0.91 in orange lines. (**B**) Heat map clustering of the co-expressed genes above the cutoff of 0.5 validated through ProteomeHD.

The 61 annotated genes were submitted to the STRING database for the analysis of Co-expression analysis. A complete table of co-expressed genes was retrieved from STRING and a grid network was created using Cytoscape 3.6.1. The genes with co-expression score > 0.5 were shortlisted and identified as hub genes (the hub genes are highlighted in red in the Fig. 5A). The complete table of the co-expression network is presented in supplementary data and the shortlisted interactions are highlighted in Green. This list of the shortlisted genes was again fed for co-expression analysis in STRING. A heat map considering the co-regulation scores of these genes through ProteomeHD was created (Fig. 5B). The genes on the heatmap were identified as hub genes through co-expression validation. These hub genes were cross verified with the genes identified through PPI interactions. CD8A and CCL5 correlate with the PPI interaction genes and could be considered as the validated hub genes.

### 3.6 Gene-drug interactions

**Fig. 6:**
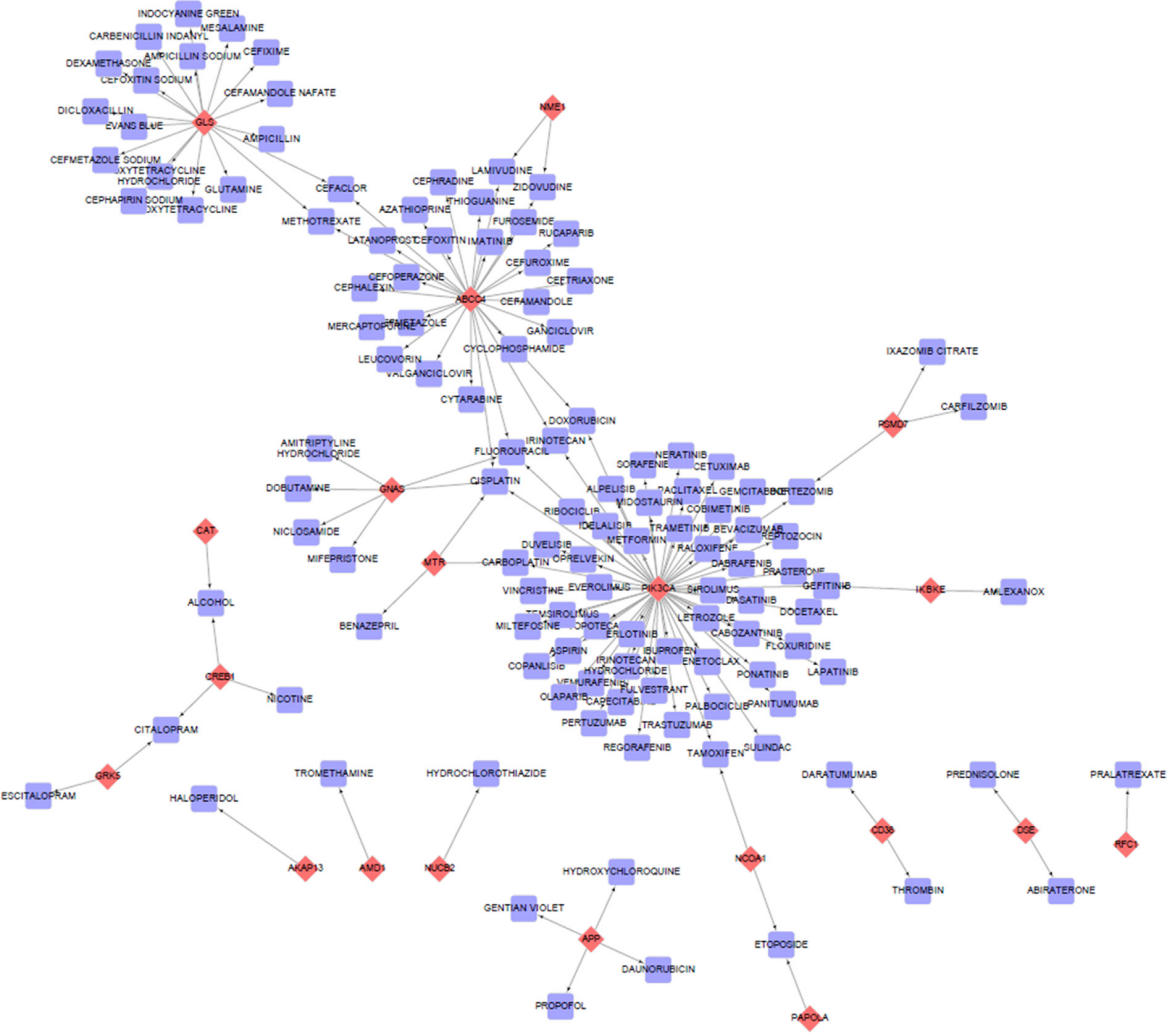
**Drug-Gene interaction network of the annotated genes through DGIdb.** The genes are highlighted in red and are in round shape whereas the drugs are highlighted in blue with rectangle shape.

The list of annotated genes was subjected to gene-drug association studies in the DGIdb, The Drug Gene Interaction Database (Sherman et al., 2007). With a set filter of approved drugs, the 61 query genes were searched against 22 source databases, 43 gene categories and 31 interaction types. This yielded a network of 141 nodes and 139 edges. The interactions found through DGIdb were put through Cytoscape 3.6.1 and an interaction network was created (Fig. 6). Out of the 61 query genes, only 20 were found to be affected by the drug interaction. Also, the shortlisted hub genes, CD8A and CCL5 were not found in the interaction, proving it to be a unique target gene for understanding the mechanism of type-2 diabetes mellitus. The complete list of the nodes and edges are presented in supplementary data.

### 3.7 Molecular docking

**Fig. 7:**
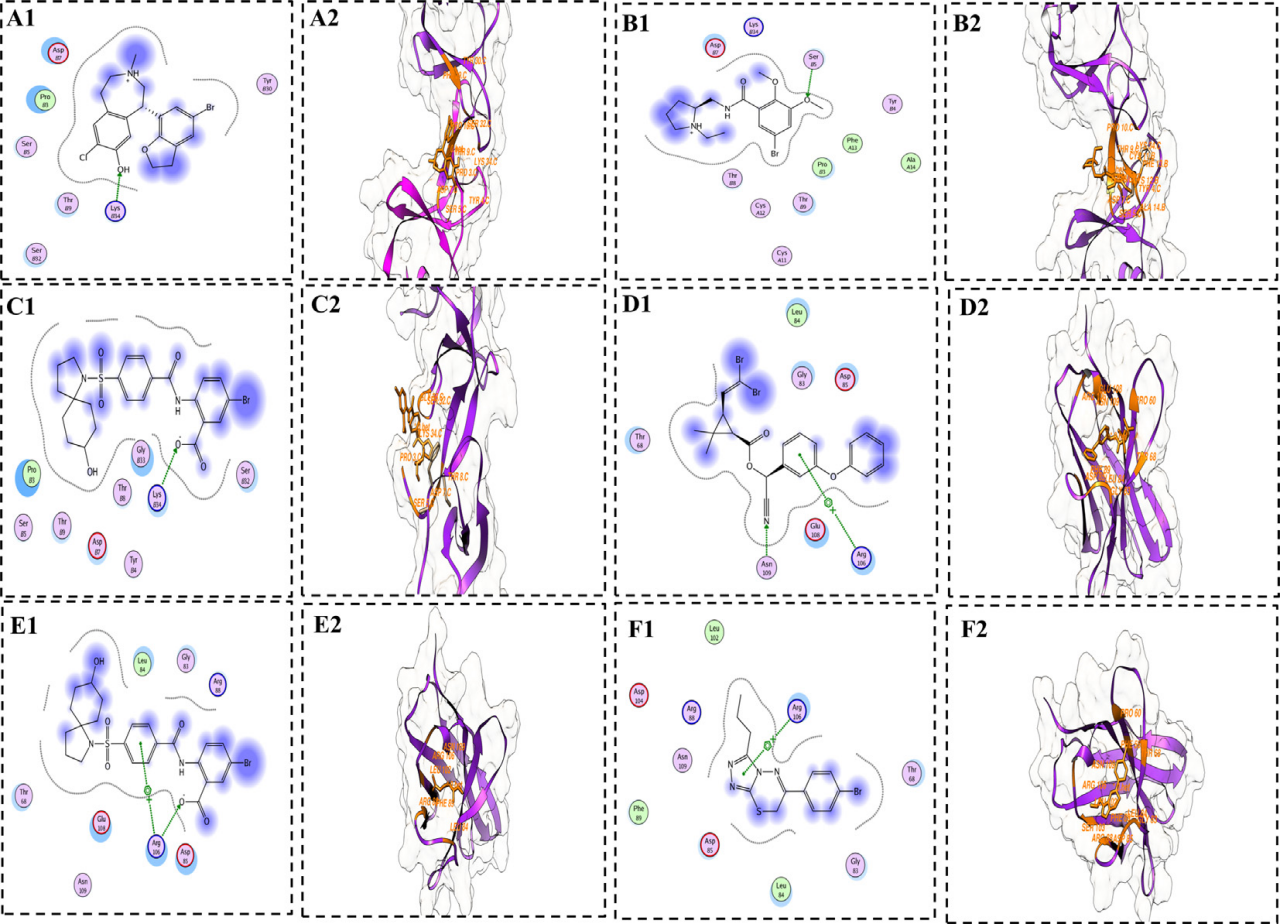
A representation of 2D and 3D interaction analysis of docked complexes. Figures with numerical value “**1**″ representing 2D interaction maps, while with “**2**” representing 3D interaction images. Green lines depicting hydrogen bindings between compounds and proteins. The figures **A-C** are representing interactions of Pubchem_CID (265), Pubchem_CID (767) and Pubchem_CID (15432541) with CCL5 Protein, respectively. The figures **D-F** are representing the interactions of Pubchem_CID (40585), Pubchem_CID (15432541) and Pubchem_CID (11227971) with CD8A protein, respectively.

Molecular docking (Suleman et al., 2021) of phytochemicals library with CCL5 (PDB ID:6AEZ) and CD8A was performed. Active site residues for both protein was predicted using the active site finder tool in MOE. After docking, screening and selection of the compounds were based on the RMSD, S-Score and hydrogen binding. Top five compounds for each protein were selected as shown in Table 3. The 2-D and 3-D interaction analysis of the selected five compounds as shown in (Fig. 7). It has been seen that Pubchem_CID (265) interacts with CCL5 protein involving hydrogen bond with LYS34 that is shown in fig A1. The figure B1 representing the interaction of Pubchem_CID (767) with CCL5 via hydrogen bond involving SER5. The figure C1 showing the interactions of Pubchem_CID (15432541) with CCL5 involving hydrogen bond with LYS34. The figure D1 showing the interactions of Pubchem_CID (40585) with CD8A through hydrogen bond with residue ASN209 and pi-hydrogen bonding with ARG106. The figure E1 represents Pubchem_CID (15432541) with CD8A via hydrogen bond ASN209 and pi-hydrogen bonding with ARG106. The figure F1 representing the interactions of CD8A with Pubchem_CID (11227971) with involved pi-pi bond with residue ARG106. The docking score ranges from −10.21 to −6.87 kcal/mol.

**Table 3:** Docking results top selected compounds after screening the phytochemicals library.

Protein	PubChem CID	S score	RMSD
CCL5	265	−10.21	0.68
	767	−10.67	1.65
	15432541	−9.06	1.28
	11417878	−8.12	1.52
	11787114	−9.93	0.95
CD8A	40585	−7.21	0.76
	15432541	−9.86	1.81
	11227971	−8.43	1.80
	109453	−7.87	1.38
	5281553	−6.87	1.27

### 3.8 Molecular dynamics simulation of top ranked compounds

#### 3.8.1 MD simulation of CCL5 with natural compound PubChem_ID:265

**Fig. 8:**
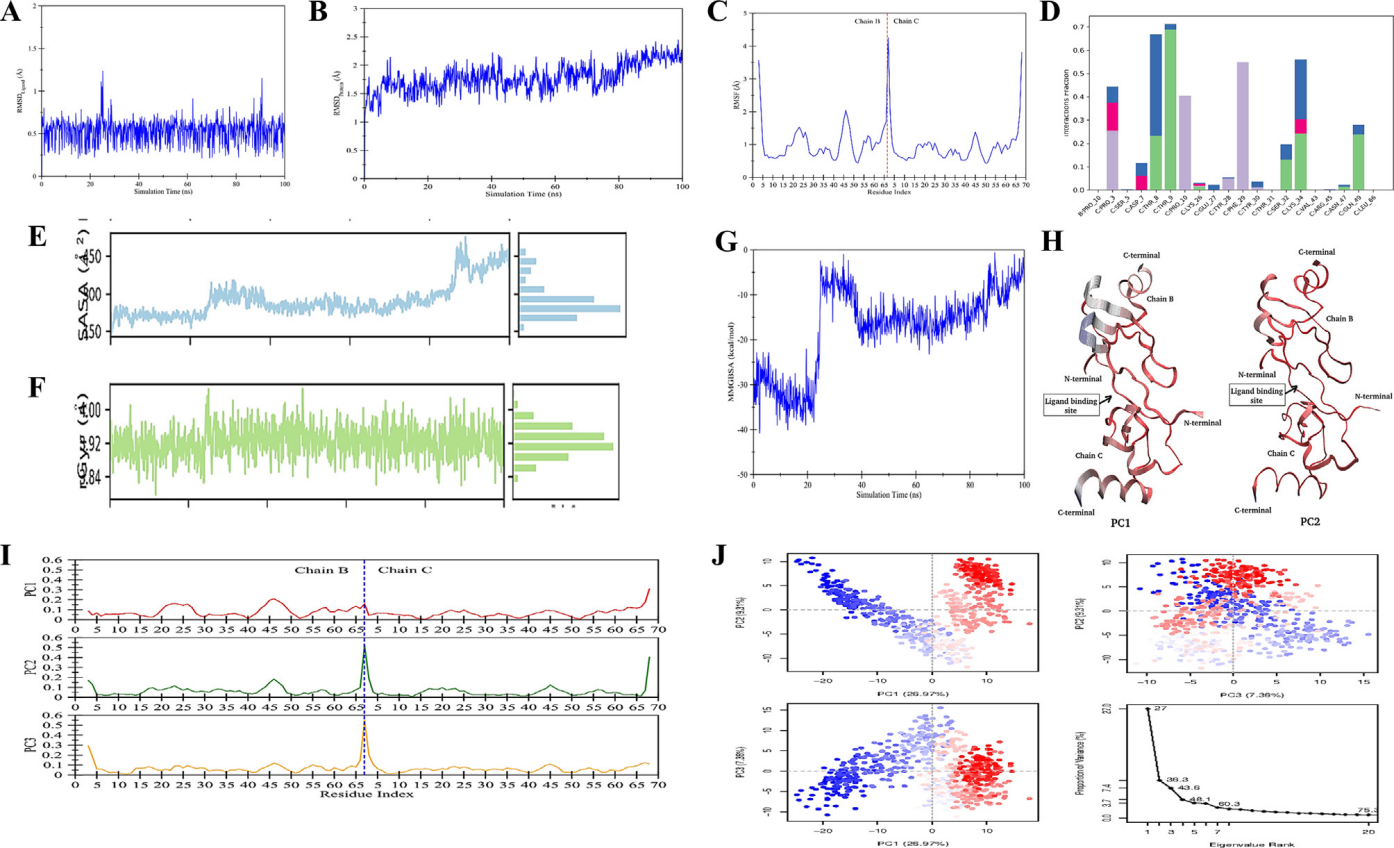
MD simulation interaction diagrams of 100 ns trajectory showing (**A**) RMSD of Ligand; (**B**) RMSD of Protein; (**C**) Root mean square fluctuation (RMSF); (**D**) Protein-ligand contacts histogram; (**E**) radius of gyration of protein; (**F**) SASA; (**G**) The Binding energy of protein–ligand complex; (**H**) mode of motion as obtained from PCA1and PCA2; (**I**) contribution of each protein residue to the first three principal components; (**J**) PCA for complex recorded from Bio3D program of R.

The MD simulation (Alamri et al., 2021) of CCL5 with compound 265 was executed with the Desmond Simulation Package for 100 ns per complex. The stability of the model-proteins were scrutinized by plotting the root mean square deviation (RMSD), assorted for protein and ligand during the production cycle as shown in Fig. 8A and 8B respectively. The RMSD for the complex showed the compact fluctuation falling at average of 0.5 s and the stable trajectory throughout the production run with maximum deviation in the range of 0.25 Å and 1.25 Å (Fig. 8A). However, for the ligand the trajectory tends to fluctuate in the span of 1 Å and 2 Å highlighting the stability as it lies below the 3 Å (Fig. 8B). To evaluate the flexibility of individual amino acid residues RMSF analysis was done for the complex that displayed RMSF value between 0.5 and 2.2 Å with the local ligand-contact fluctuation at several points with extreme at residues (45–50) for both the chain as shown in Fig. 8C. The convolutions were in the suitable range corresponding of protein and ligand.

The interaction profiles for the selected complex were computed as is shown in Fig. 8D. The complex established interaction with THR_9 and GLN_49 via H bonding PHE_29, and PRO_10 through the formation of a hydrophobic bond shown in Fig. 8D. Further, the parameter such as radius of gyration (Kustatscher et al.,) for the complex calculated through the simulation of 100 ns was 0.1 Å ± 0.5 Å (Fig. 8E). The sustained trend in trajectory depicts the compactness throughout the interaction. SASA values were assessed to know the modification in the conformational setup at 100 ns that revealed to be 0.2 ± 0.3 Å (Fig. 8F).

The MMGBSA (Rehan Khalid et al., 2018) recorded in the span of 100 ns for evaluation of inhibitor associating in the respective binding pocket to monitor the difference in binding energies during the run. The Fig. 8G demonstrate the trend that encourages reliability of current analysis regarding free energy that tend to increase following 22 ns and displayed minor episode of upregulation and decline.

Moreover, the modes of motion of the protein elaborated by the first and second PC of both chains are represented in Fig. 8H. These representations of structures encourage the mobility in independent manner of N-terminal and C-terminal domains that signify the opening potential as well as closing of the pocket. The contribution of each protein residue to the first three principal components is recorded as shown in Fig. 8I. The Principal Component Analysis (PCA) (Muneer et al., 2019) was recorded by the first three principal components (PCs). All three PCs captured displayed values as 26.97%, 93.1% and 7.36% of structural variance in complex respectively (Fig. 8J).

#### 3.8.2 MD simulation of CD8A with 40585

**Fig. 9:**
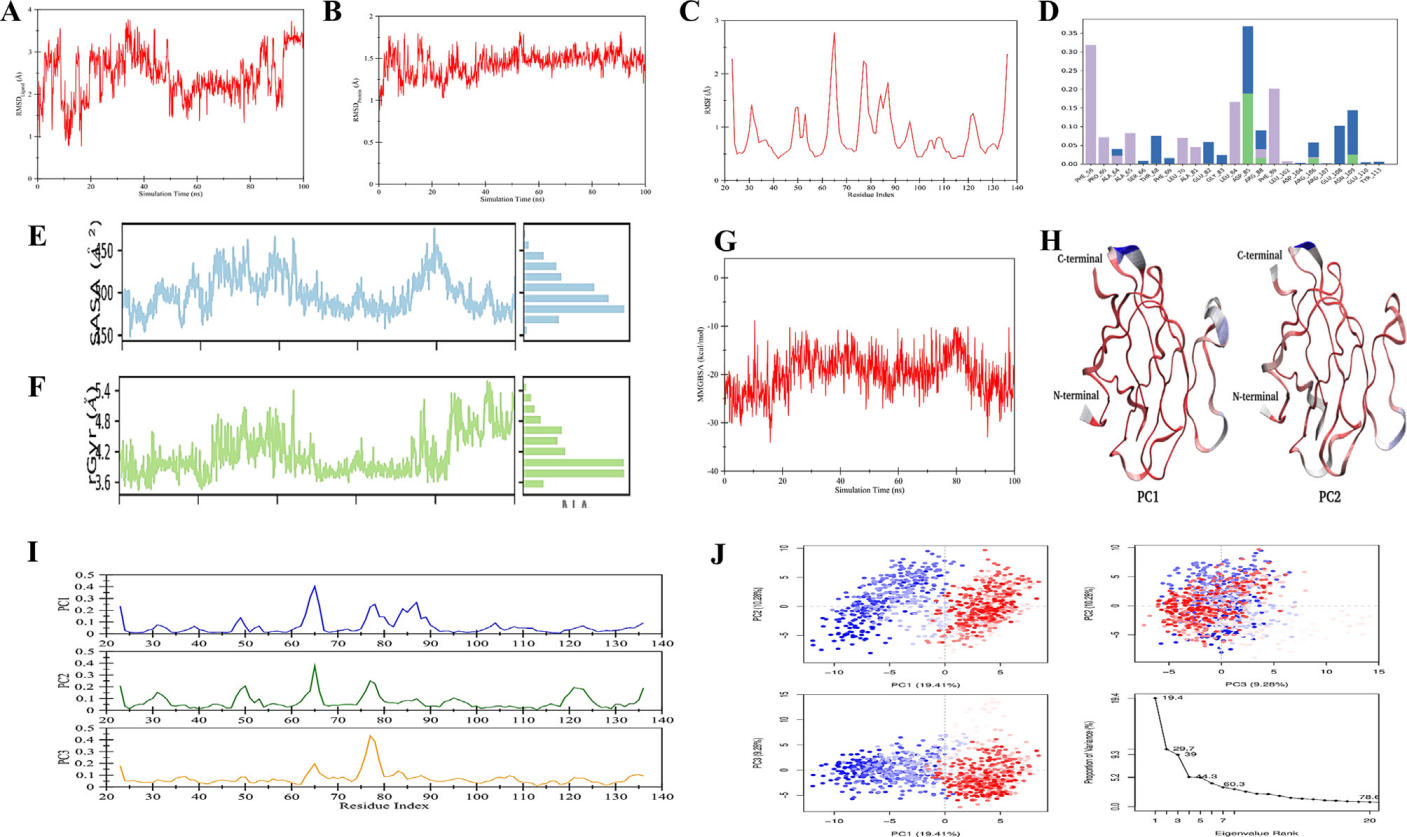
MD simulation interaction diagrams with 100 ns trajectory showing (**A**) RMSD of Ligand; (**B**) RMSD of Protein; (**C**) Root mean square fluctuation; (**D**) Protein-ligand contacts histogram; (**E**) The radius of gyration of protein; (**F**) SASA; (**G**) The binding energy of protein–ligand complex; (**H**) mode of motion as obtained from PCA1and PCA2; (**I**) contribution of each protein residue to the first three principal components; (**J**) PCA for complex recorded from Bio3D program of R.

The MD simulation of CD8A was recorded with the Desmond Simulation Package for 100 ns per complex. The scrutinizing of the stability of complex RMSD plot assorted for protein and ligand were recorded during the production run as shown in Fig. 9A and 9B respectively. The RMSD for the complex displayed several episodes of fluctuation at 15 ns and 75 ns with an increase in trajectory in between at 40 ns (Fig. 9A). For the ligand, the fluctuation was in the span of 1 Å and 1.75 Å in the acceptable range that is below 3 Å (Fig. 9B). The RMSF recorded that ranges between 0.5 and 2.5 Å with the local ligand-contact fluctuation reaching extreme at multiple positions of residues including with extreme at residues (60–70) and (75–80) as shown in Fig. 9C. The suitable convolutions regarding protein and ligand displayed the compactness.

The interaction profiles computed in Fig. 9D showed that formation of water bridge involves interaction with residues GLU_108. The hydrophobic interactions were established by PHE_58, PRO_60, LEU_84 and PHE_89. Additionally, parameters such as radius of gyration (Kustatscher et al.,) recorded through for 100 ns was 0.2 Å ± 0.4 Å (Fig. 9E). SASA values were revealed to be 0.1 ± 0.3 Å (Fig. 9F). The trend was revealed to be similar as evident from trajectory thereby strengthening the compactness in interaction.

The MMGBSA recorded in the span of 100 ns for evaluation of inhibitor binding in the corresponding pocket to observe the difference in binding energies in the simulation. The reliability of current analysis is supported by free energy recorded that displayed consistency with average of 20 (kcal/mol) (Fig. 9G). The modes of motion of complex by the first and second PC of both chains are represented in Fig. 9H. These structures support the independent mobility at terminal N and C of domains that specify potential of plasticity within the pocket. The contribution of each protein residue to the first three principal components is highlighted in Fig. 9I. The PCA for the three PCs displayed values of 19.41%, 10.28% and 9.28% in complex respectively as is highlighted in Fig. 9J.

## 4 Discussion

T2DM is the most common disorder observed among all kinds of population across the globe and is a significant public health concern (Chen et al., 2012). In the current scenario with currently available medical expertise, diabetes is a permanent disorder. There is always an ever-growing demand for effective diagnostic tools and therapeutic markers (Kustatscher et al., 2019). The potential predictive biomarkers of the diabetic peripheral neuropathy were identified (Kustatscher et al., 2019)*.* The gene expression profiles from GEO database to identify the molecular mechanisms and also the key hub genes contributing to synovial inflammation with existing conditions of rheumatoid arthritis were also studied (Kustatscher et al., 2019).

The causative genes, their elemental participation, the underlying pathways leading to the condition and the pathogenicity of T2DM is the need of the hour and several permutations and combinations would pave the way in targeting the disease (Kumar et al., 2015). In this direction, identification of hub genes through integrated bioinformatics approaches has been successful. Differential Gene Analysis (DEG) is performed with Gene ontology analysis (Gene ontology and reactome) for GSE25724 and GSE101931 retrieved from GEO-NCBI. Protein-Protein Interactions (PPI) resulted in a long list of hub genes that could possibly be causative gene elements.

The annotated genes were mapped as protein-coding genes and were submitted to the STRING database for the identification of the interactions. A full STRING network with a medium confidence score was set to 0.40 and the 0.05 FDR. The hub genes were analyzed using Cytoscape 3.6.1. The network was analyzed using the network analyzer to discover the network parameters. The size of the nodes was subjected to the increasing average shortest path length and the size of the edges were subjected to the interaction score provided by the STRING database.

The intensity and frequency of occurrence of T2DM varies with populations and subgroups. It is often associated with a strong unclear genetic predisposition (Association 2009). Co-expression network analysis was used to scale down the number of genes using the STRING database, and Drug-Gene Interactions. It is understood that the progression of the condition might be due to the genes that alter the underlying pathways. The identified hub genes, CD8A and CCL5 were not found in the interaction, hence they are the key genes responsible for the mechanism of the condition (Zhou et al., 2014).

The CD8 antigen is a cell surface glycoprotein found on most cytotoxic T lymphocytes that mediates efficient cell–cell interactions within the immune system. CD8A (CD8a Molecule) is a protein coding gene. The CCL5 (C-C Motif Chemokine Ligand 5) gene is one of the several chemokine genes clustered on the q-arm of chromosome 17 and is a protein coding gene. Many studies suggest that an increase in serum levels of CCL5 occurs in T2DM and are closely related to postprandial hyperglycemia (Dworacka et al., 2014).

In spite of the fact that docking studies have been effectively utilized in computing the limiting postures of ligands for some proteins, they refrained in evaluating ligand potential to binding. The protein rigidity in consideration overlook the conformational modification in the course of docking of certain ligands (Hassan et al., 2002). The modification in conformation during protein–ligand connections could be contemplated during MD trajectory reproductions. The conformity changes in interaction of protein–ligand have been comprehensively examined through MD simulation strategies.

With substantial proliferation of computational biology and utilizing bioinformatics tools have forced stringent compliance in drug designing, thus helps reduction in the expenses and turn over time needed for in vivo screening and experimentations prerequisite for successful drug development (Barh et al., 2011). These approaches have culminated lab including drug candidates recognition, structure-based designing and screening of the drug molecule, genome-based investigation to locate host-specific targets (Lakshmi and Ramyachitra 2020).

Recently, computational skills determined the probability of linking various molecules before their development and estimation in experimental setup. Thus, the top-ranked compounds further chosen for MD simulation which demonstrated outcomes within the acceptable range of parameters proposes the interaction results are reliable in conformation. Nonetheless, investigative efforts on drug-gene interactions in our work, potentially inspire the research activities to identify therapeutic drug candidates for T2DM.

## 5 Conclusion

In the current work, a new mechanism was proposed which explains the progression in pathogenesis of T2DM that might be due to the genes that disturb the pathways leading to the disease condition. The shortlisted hub genes, CD8A and CCL5 were not found in the interaction, proving it to be a unique target gene for understanding the mechanism of T2DM, hence these genes might act as potential biomarkers for diagnosis of T2DM at an early stage. The docking and simulation done proposes the candidate complex to be compact and stable. The hub genes cause disruption in cellular pathways which deeply worsens the disease condition. Experimental models may be effectively designed to aid the detection of pathogenesis, evaluation of risk, and in determining the targeted therapies with reference to the hub genes.

## Declaration of Competing Interest

The authors declare that they have no known competing financial interests or personal relationships that could have appeared to influence the work reported in this paper.

## References

[b0005] Ahmed B., Ashfaq U.A., Ul Qamar M.T., Ahmad M. (2014). Anti-cancer potential of phytochemicals against breast cancer: Molecular docking and simulation approach. Bangladesh J. Pharmacol..

[b0010] Alamri M.A., ul Qamar M.T., Afzal O., Alabbas A.B., Riadi Y., Alqahtani S.M. (2021). Discovery of anti-mers-cov small covalent inhibitors through pharmacophore modeling, covalent docking and molecular dynamics simulation. J. Mol. Liq..

[b0015] Altharawi A., Ahmad S., Alamri M.A., ul Qamar M.T. (2021). Structural insight into the binding pattern and interaction mechanism of chemotherapeutic agents with sorcin by docking and molecular dynamic simulation. Colloids Surf. B: Biointerf..

[b0020] Ameer O. (2011). An ethnopharmacological and phytochemical investigation.

[b0025] Amos S., Akah P.A., Binda L., Enwerem N.M., Ogundaini A., Wambebe C., Hussaini I.M., Gamaniel K.S. (2003). Hypotensive activity of the ethanol extract of pavetta crassipes leaves. Biol. Pharm. Bull..

[b0030] Arif R., Ahmad S., Mustafa G., Mahrosh H.S., Ali M., Tahir ul Qamar M., Dar H.R. (2021). Molecular docking and simulation studies of antidiabetic agents devised from hypoglycemic polypeptide-p of momordica charantia. BioMed Res. Int..

[b0035] Association A.D. (2009). Diagnosis and classification of diabetes mellitus. Diabetes Care.

[b0040] Barh D., Tiwari S., Jain N., Ali A., Santos A.R., Misra A.N., Azevedo V., Kumar A. (2011). In silico subtractive genomics for target identification in human bacterial pathogens. Drug Develop. Res..

[b0045] Barrett T., Troup D.B., Wilhite S.E., Ledoux P., Rudnev D., Evangelista C., Kim I.F., Soboleva A., Tomashevsky M., Marshall K.A. (2009). Ncbi geo: Archive for high-throughput functional genomic data. Nucleic Acids Res..

[b0050] Barrett T., Wilhite S.E., Ledoux P., Evangelista C., Kim I.F., Tomashevsky M., Marshall K.A., Phillippy K.H., Sherman P.M., Holko M. (2012). Ncbi geo: Archive for functional genomics data sets—update. Nucleic Acids Res..

[b0055] Berry N.T., Hubal M., Wideman L. (2018). The effects of an acute exercise bout on gh and igf-1 in prediabetic and healthy african americans: A pilot study investigating gene expression. PLoS ONE.

[b0060] Brechot C., Kremsdorf D., Soussan P., Pineau P., Dejean A., Paterlini-Brechot P., Tiollais P. (2010). Carcinogenese hepatique induite par le virus de l'hepatite b: Un modele de carcinogenese viro-induite chez l'homme. Pathol. Biol. (Paris).

[b0065] Carey E., Leighton J., Shiff A., Heigh R., Sharma V., Fleischer D. (2004). The value of capsule endoscopy for evaluation of abdominal pain and/or diarrhea. Gastroenterology.

[b0070] Chang J.-W., Ding Y., Qamar M.Tahir ul, Shen Y., Gao J., Chen L.-L. (2019). A deep learning model based on sparse auto-encoder for prioritizing cancer-related genes and drug target combinations. Carcinogenesis..

[b0075] Chen L., Magliano D.J., Zimmet P.Z. (2012). The worldwide epidemiology of type 2 diabetes mellitus—present and future perspectives. Nat. Rev..

[b0080] Dedov I., Shestakova M., Benedetti M.M., Simon D., Pakhomov I., Galstyan G. (2016). Prevalence of type 2 diabetes mellitus (t2dm) in the adult russian population (nation study). Diabetes Res. Clin. Pract..

[b0085] Demchak B., Hull T., Reich M., Liefeld T., Smoot M., Ideker T., Mesirov J.P. (2014). Cytoscape: The network visualization tool for genomespace workflows. F1000Res..

[b0090] Donadon V., Balbi M., Casarin P., Vario A., Alberti A. (2008). Association between hepatocellular carcinoma and type 2 diabetes mellitus in italy: Potential role of insulin. World J. Gastroenterol.: WJG.

[b0095] Dong G., Qu L., Gong X., Pang B., Yan W., Wei J. (2019). Effect of social factors and the natural environment on the etiology and pathogenesis of diabetes mellitus. Int. J. Endocrinol..

[b0100] Dworacka M., Krzyżagórska E., Iskakova S., Bekmukhambetov Y., Urazayev O., Dworacki G. (2014). Increased circulating rantes in type 2 diabetes. Eur. Cytokine Netw..

[b0105] Fabregat A., Sidiropoulos K., Garapati P., Gillespie M., Hausmann K., Haw R., Jassal B., Jupe S., Korninger F., McKay S. (2016). The reactome pathway knowledgebase. Nucleic Acids Res..

[b0110] Freshour S.L., Kiwala S., Cotto K.C., Coffman A.C., McMichael J.F., Song J.J., Griffith M., Griffith O.L., Wagner A.H. (2021). Integration of the drug–gene interaction database (dgidb 4.0) with open crowdsource efforts. Nucleic Acids Res..

[b0115] Griss J., Viteri G., Sidiropoulos K., Nguyen V., Fabregat A., Hermjakob H. (2020). Reactomegsa-efficient multi-omics comparative pathway analysis. Mol. Cell. Proteomics.

[b0120] Hassan M.M., Hwang L.-Y., Hatten C.J., Swaim M., Li D., Abbruzzese J.L., Beasley P., Patt Y.Z. (2002). Risk factors for hepatocellular carcinoma: Synergism of alcohol with viral hepatitis and diabetes mellitus. Hepatology.

[b0125] Hsiang J.C., Gane E.J., Bai W.W., Gerred S.J. (2015). Type 2 diabetes: A risk factor for liver mortality and complications in hepatitis b cirrhosis patients. J. Gastroenterol. Hepatol..

[b0130] Khan W., Ashfaq U.A., Aslam S., Saif S., Aslam T., Tusleem K., Maryam A., ul Qamar M.T. (2017). Anticancer screening of medicinal plant phytochemicals against cyclin-dependent kinase-2 (cdk2): An in-silico approach. Adv. Life Sci..

[b0135] Kumar N.P., Sridhar R., Nair D., Banurekha V.V., Nutman T.B., Babu S. (2015). Type 2 diabetes mellitus is associated with altered cd 8+ t and natural killer cell function in pulmonary tuberculosis. Immunology.

[b0140] Kustatscher G., Grabowski P., Schrader T.A., Passmore J.B., Schrader M., Rappsilber J. (2019). Co-regulation map of the human proteome enables identification of protein functions. Nat. Biotechnol..

[b0145] Lakshmi P., Ramyachitra D. (2020). Statistical modelling and machine learning principles for bioinformatics techniques, tools, and applications.

[b0150] Mi H., Muruganujan A., Ebert D., Huang X., Thomas P.D. (2019). Panther version 14: More genomes, a new panther go-slim and improvements in enrichment analysis tools. Nucleic Acids Res..

[b0155] Muneer I., Ahmad S., Naz A., Abbasi S.W., Alblihy A., Aloliqi A.A., Alkhayl F.F.A., Alrumaihi F., Ahmad S., El Bakri Y. (2021). Discovery of novel inhibitors from medicinal plants for v-domain ig suppressor of t-cell activation. Front. Mol. Biosci..

[b0160] Muneer I., Tusleem K., Abdul Rauf S., Hussain H.M., Siddiqi A.R. (2019). Discovery of selective inhibitors for cyclic amp response element-binding protein: A combined ligand and structure-based resources pipeline. Anti-cancer Drugs.

[b0165] Rehan Khalid R., Tahir ul Qamar M., Maryam A., Ashique A., Anwar F., Geesi M.H., Siddiqi A.R. (2018). Comparative studies of the dynamics effects of bay60-2770 and bay58-2667 binding with human and bacterial h-nox domains. Molecules..

[b0170] Sherman B.T., Tan Q., Collins J.R., Alvord W.G., Roayaei J., Stephens R., Baseler M.W., Lane H.C., Lempicki R.A. (2007). The david gene functional classification tool: A novel biological module-centric algorithm to functionally analyze large gene lists. Genome Biol..

[b0175] Sudesna C., Khunti K., Davies M.J. (2017). Type 2 diabetes. Lancet.

[b0180] Sufyan M., Ashfaq U.A., Ahmad S., Noor F., Saleem M.H., Aslam M.F., El-Serehy H.A., Aslam S. (2021). Identifying key genes and screening therapeutic agents associated with diabetes mellitus and hcv-related hepatocellular carcinoma by bioinformatics analysis. Saudi J. Biol. Sci..

[b0185] Suleman M., ul Qamar M.T., Shoaib Saleem S.A., Ali S.S., Khan H., Akbar F., Khan W., Alblihy A., Alrumaihi F., Waseem M. (2021). Mutational landscape of pirin and elucidation of the impact of most detrimental missense variants that accelerate the breast cancer pathways: A computational modelling study. Front. Mol Biosci..

[b0190] Szklarczyk D., Gable A.L., Nastou K.C., Lyon D., Kirsch R., Pyysalo S., Doncheva N.T., Legeay M., Fang T., Bork P. (2021). The string database in 2021: Customizable protein–protein networks, and functional characterization of user-uploaded gene/measurement sets. Nucleic Acids Res..

[b0195] Tahir ul Qamar M., Maryam A., Muneer I., Xing F., Ashfaq U.A., Khan F.A., Anwar F., Geesi M.H., Khalid R.R., Rauf S.A. (2019). Computational screening of medicinal plant phytochemicals to discover potent pan-serotype inhibitors against dengue virus. Rep. Sci..

[b0200] Tahir ul Qamar M., Mirza M.U., Song J.-M., Rao M.J., Zhu X., Chen L.-L. (2021). Probing the structural basis of citrus phytochrome b using computational modelling and molecular dynamics simulation approaches. J. Mol. Liq..

[b0205] ul Qamar M.T., Ahmad S., Khan A., Mirza M.U., Ahmad S., Abro A., Chen L.-L., Almatroudi A., Wei D.-Q. (2021). Structural probing of hapr to identify potent phytochemicals to control vibrio cholera through integrated computational approaches. Comput. Biol. Med..

[b0210] Zhou H.-B., Hu J.-Y., Hu H.-P. (2014). Hepatitis b virus infection and intrahepatic cholangiocarcinoma. World J. Gastroenterol.: WJG.

